# Supplementary figures and images for: Exploring barriers to accessing healthy diets among pregnant women living with HIV in the Njombe region, Tanzania: A qualitative study
Source: PLOS Glob Public Health. 2025 Oct 10;5(10):e0004438. doi: 10.1371/journal.pgph.0004438 (PMC12513609; doi:10.1371/journal.pgph.0004438)

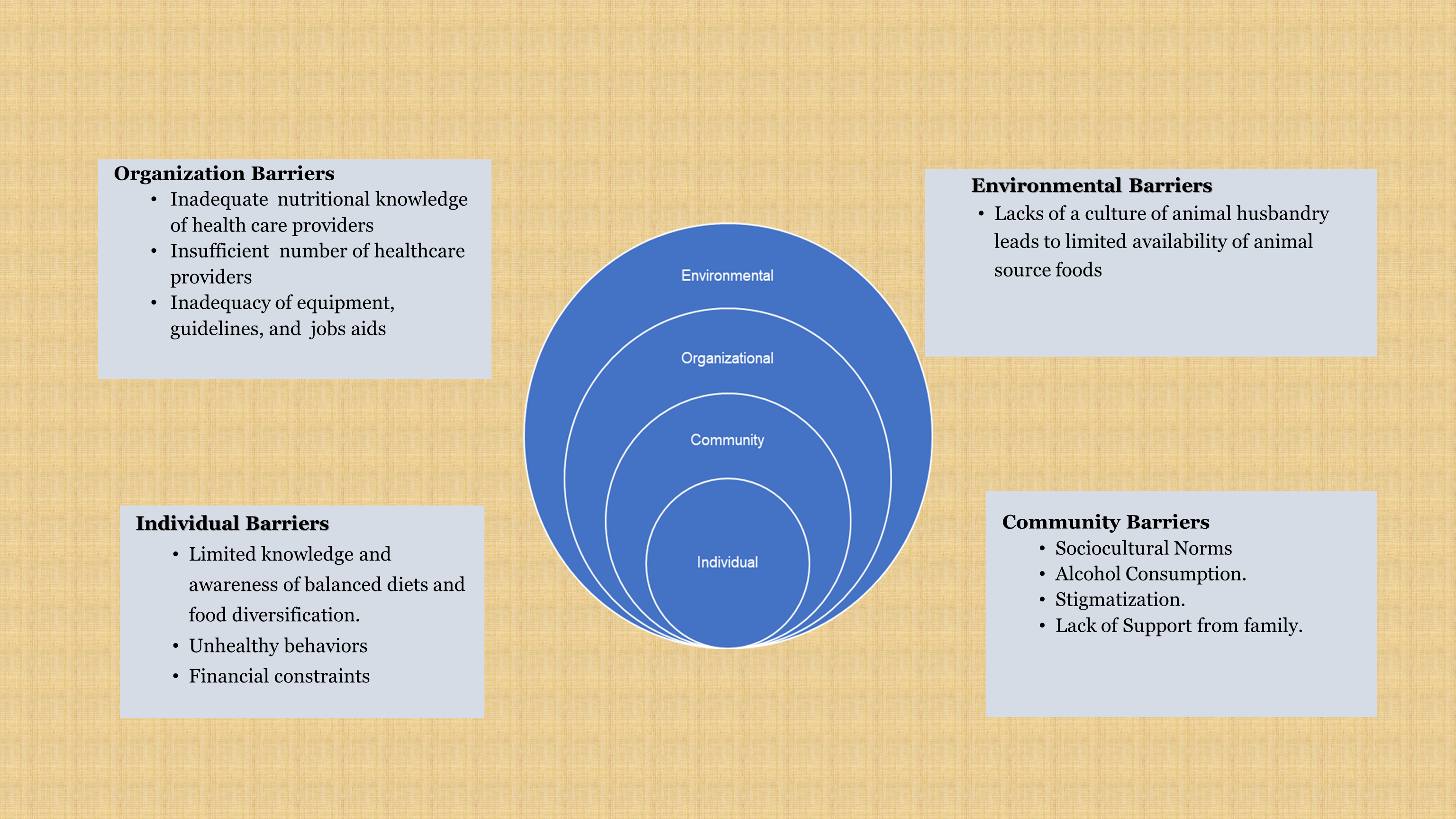

Supplement: S1 Fig — (TIF) [file pgph.0004438.s001.tif]
