# Supplementary material for: Exploring barriers to accessing healthy diets among pregnant women living with HIV in the Njombe region, Tanzania: A qualitative study
Source: PLOS Glob Public Health. 2025 Oct 10;5(10):e0004438. doi: 10.1371/journal.pgph.0004438 (PMC12513609; doi:10.1371/journal.pgph.0004438)
Supplement: S1 Text — (DOC) [file pgph.0004438.s002.doc]

***S1 FGD interview guide***

Focus Group Discussion Interview Guide for HIV Pregnant women

1. What kind of foods is normally consumed by pregnant women in your area? What are the things that push them to eat those foods? Probe;
   1. Do pregnant women eat the same amount of foods like other members in the family? Why? (Ask about consumption of specific types of foods in relation to; religious beliefs, ethnicity related, traditional beliefs, income/food prices, availability of foods, seasonability, advise from health care providers, advise from partners and in-laws).
   2. Do pregnant women eat the same number of meals like other members in the family? Why (Ask and probe as above).
2. Please tell us the foods/things that are not advised to be consumed during pregnancy? Why Probe; alcohol, soil, use of tobacco, charcoal etc.( Ask for reasons; religious beliefs, traditional beliefs, cultural norms, advise from health care providers, in-laws, parents, relatives, friends etc.,
3. What decisions do you have about food consumption in your household? Probe; At the household level, who makes the final decisions about the type of foods to be cooked, number of meal to be consumed? How does this affect consumption of foods for pregnant women?
4. Where do you get information or advise about consumption of foods during pregnancy? Probe media, social media, friends who are pregnant or have been pregnant, elderly women, partners, health care providers, traditional birth attendants.
5. What eating habits do you think are appropriate for HIV pregnant women? (Probe for types of foods, number of meals, snacks, quantity of food consumed, and use of supplements (FEFO). Probe why woman should change her diets when she finds out pregnant?
6. Please tell us about status of stigma to HIV pregnant women. Probe; stigma from community, family and health care providers etc.? Ask about discrimination that affects HIV pregnant women to access healthy diet. Ask how they are stigmatized and discriminated; eating separately from other members of the family, giving small amount of foods, using separate serving dishes, glasses, cups, plates, discrimination in purchasing foods. How does this kind of stigma affect nutrition of HIV pregnant women?
7. What are the things that enable HIV pregnant women to practice healthy eating? (Ask why the mentioned reasons are the major ones) high price of the foods, traditional and cultural beliefs, distance from and to health facilities, availability of the foods, availability of health services, availability of nutritional supplements, capacity of health care providers in providing nutrition education, number of health care providers.
8. Probe; What do you think are the major barriers for HIV pregnant women to access healthy diet? (Probe for reasons why the mentioned barriers are the major one, Probe for poverty, high price of foods, traditions/taboos, no time to prepare food due to workload, long distance to the health facility, low capacity of health care providers in providing nutrition education, unavailability of foods, lack of support from partner/husband and any others).
9. What should be done to enable HIV pregnant women to access healthy diets? (Probe nutrition education and counseling, economic empowerment, engage partners to provide advice to pregnant women, engagement of other social support groups (TASAF).
