# Supplementary material for: Exploring barriers to accessing healthy diets among pregnant women living with HIV in the Njombe region, Tanzania: A qualitative study
Source: PLOS Glob Public Health. 2025 Oct 10;5(10):e0004438. doi: 10.1371/journal.pgph.0004438 (PMC12513609; doi:10.1371/journal.pgph.0004438)
Supplement: S2 Text — (DOC) [file pgph.0004438.s003.doc]

***S2*** ***KII guide***

**KEY INFORMAT INTERVIEW (KII) GUIDE**

1. Please tell us about your experience in this section related to nutrition for pregnant women. Ask what is your role in improving HIV pregnant woman health and Nutrition? Probe (existing nutrition program) what are the priorities for improving nutrition for HIV pregnant women? Probe what efforts do you take to ensure that HIV pregnant women access and consume nutritious foods? Probe availability of FEFOL in relation to number of pregnant women in the health facilities, How do you participate in increasing community awareness in order to help HIV pregnant women to eat healthy diets? Probe for available job aids tools used to raise awareness ( Guides, posters and anthropometric equipment)
2. What kind of foods should HIV-pregnant women consume in large quantities? Ask Why. What types of foods are prohibited during pregnancy or not allowed to be consumed during pregnancy? Ask: How does information related to nutrition for HIV pregnant women reach health care providers or the community? (Training, media, social media, IEC materials, health care providers, CHWs, etc.).
3. Please tell us about status of stigma to pregnant women living with HIV. Probe; stigma from community, family and health care providers etc.? Ask about how discrimination affects HIV pregnant women to access healthy diet.
4. What are the things that enable HIV pregnant women to practice healthy eating? (Ask why the mentioned reasons are the major ones) high price of the foods, traditional and cultural beliefs, distance from and to health facilities, availability of the foods, availability of health services, availability of nutritional supplements, capacity of health care providers in providing nutrition education, number of health care providers.

Supporting in food preparation from close relative, partners, supporting in household Probe; what do you think are the major barriers for HIV pregnant women to access healthy diet? (Probe for reasons why the mentioned barriers are the major one, Probe for poverty, high price of foods, traditions/taboos, no time to prepare food due to workload, long distance to the health facility, low capacity of health care providers in providing nutrition education, unavailability of foods, lack of support from partner/husband and any others).

1. What should be done to enable HIV pregnant women to access healthy diets? (Probe for proposed nutrition counselling and education provided at health facility, Home visits, Village health day, community outreach and others).
