# Supplementary material for: Exploring barriers to accessing healthy diets among pregnant women living with HIV in the Njombe region, Tanzania: A qualitative study
Source: PLOS Glob Public Health. 2025 Oct 10;5(10):e0004438. doi: 10.1371/journal.pgph.0004438 (PMC12513609; doi:10.1371/journal.pgph.0004438)
Supplement: S3 Text — (DOC) [file pgph.0004438.s004.doc]

**S3 Thematic and Key F**actors

| **Theme** | **Sub-Theme** | **Key Factors** | **Summary** |
| --- | --- | --- | --- |
| Individual Factors | Knowledge of nutritious diets | Inadequate knowledge about a balanced diet | Pregnant women living with HIV have limited knowledge of what constitutes diets; diets rely on staple foods with little diversity |
|  | Unhealthy behaviors regarding nutritious diets | Behaviors that result in pregnant women not getting enough nutrients for a better pregnancy outcome | craving for non-nutritive items(geophagy), displacing nutrient-rich foods in the diet, may have adverse implications for the overall health of HIV pregnant women |
|  | Financial Constraints | Limited financial constraints affecting access to diversified foods | Limited income restricts the ability to afford diverse and nutritious foods. |
| Community Factors | Sociocultural norms | Influence of traditional beliefs on dietary choices | Traditional beliefs restrict consumption of certain nutritious foods, e.g., eggs are avoided to prevent hairless babies |
|  | Food restrictions and Alcohol consumption | Habits that hinder consumption of nutritious diet during pregnancy | Pregnant women are generally allowed to consume most foods, but alcohol use during pregnancy persists despite restrictions, impacting diet and health |
|  | Stigmatization | General social life and acquisition of health services stigmatization and how it affects access to healthy diets | Generally, stigma has decreased but persists at individual and family levels, leading to secrecy and stress which in turn affect nutrition. |
|  | The influence and support of spouses | Emotional and financial support that affects HIV pregnant women to access and consume healthy diets | Emotional, financial and practical support from spouses is limited; leading to heavy workloads that limit time for healthy eating |
| Organizational Factors | Nutrition education | Unavailability and inconsistency of community nutrition education | Community nutrition education programs exist but inconsistent and limited |
|  | Knowledge and skills of Healthcare Providers | Inadequate health care providers with knowledge and skills on nutrition | Shortage of trained health care providers and limited refresher training hinder provision of quality nutrition services |
|  | Availability of health care providers | Inadequate health care providers providing health and nutrition services | Health facilities exist near communities but often have few providers and long waits |
|  | Availability of Working Tools | Inadequate essential working tools for assessing health and nutritional status | Essential equipment is mostly available but new facilities lack some basic tools like weighing scales |
|  | Proximity to Healthcare Services | Accessibility of healthcare services for HIV pregnant women | All the study districts have a sufficient number of facilities located near their village |
| Environmental/contextual factors | *Food availability and Seasonality* | Limited availability of nutritious foods | Ample food during rainy season and through irrigation, mostly plant-based, animal husbandry is rare, limiting availability and affordability of animal protein rich foods |
