# Supplementary material for: Exploring barriers to accessing healthy diets among pregnant women living with HIV in the Njombe region, Tanzania: A qualitative study
Source: PLOS Glob Public Health. 2025 Oct 10;5(10):e0004438. doi: 10.1371/journal.pgph.0004438 (PMC12513609; doi:10.1371/journal.pgph.0004438)
Supplement: Attachment — Submitted filename: Peer review report.docx [file pgph.0004438.s006.docx]

I would like to appreciate this opportunity to review the study by Shine et al., titled ‘Barriers to Accessing a Healthy Diet Among HIV-Positive Pregnant Women in the Njombe Region of Tanzania: A Qualitative Study.’ This study addresses an important issue in the field and provides valuable findings. However, before it can be considered for publication, I believe there are several key areas that require revision. Please consider the following comments to improve the manuscript.

**Title**

- “HIV pregnant women” seems not to be a standard way of referring to this group. Please consider alternatives like “HIV-positive pregnant women” or “pregnant women living with HIV”. Consequently, consider revising the title to something like:
- **Barriers to accessing adequate nutrition among pregnant women living with HIV in the Njombe region of Tanzania: A qualitative study** or
- **Exploring barriers to accessing healthy diets among pregnant women living with HIV in the Njombe region, Tanzania** or
- **Challenges to accessing healthy diets among pregnant women living with HIV in the Njombe region, Tanzania**

**Abstract**

- Correct “HIV pregnant women” to “HIV-positive pregnant women” or “pregnant women living with HIV” in the title, methods section of the abstracts, in the keywords, and in all places where it has been used.

**Introduction**

- Can you use “Diets during pregnancy” instead of “Pregnancy diets”?
- The sentence “Poor nutrition during pregnancy is linked to one-fifth of maternal deaths and an increase in adverse pregnancy outcomes [3]” would do better as an opening one.
- Avoid the term “**HIV-infected**”**.** It is generally considered outdated and potentially stigmatizing, as it defines the person by their condition in a way that may be perceived negatively. It focuses on the “infection” rather than the individual. The language around HIV has evolved to be more respectful and person-centered, with an emphasis on the individual’s status without stigmatizing them. Therefore, consider such terms as “**HIV-positive**” or “**Pregnant women living with HIV**”
- For the purpose of maintaining accuracy, “individuals living with HIV (PLHIV)” could be “people living with HIV (PLHIV)”.
- Please avoid the phrase **“Key nutritious challenges include low income, ...”** It is somewhat unclear because “nutritious” is typically used to describe food, not challenges. A better term to use would be “**nutritional”**, which specifically relates to diet and nutrition. In this case, a revised phrase would be “**Key nutritional challenges include low income, ...”**
- The authors conducted this study in the Njombe region. What were the specific reasons for selecting this region as the study area, and how does it relate to the research question?
- While the study focuses on barriers to accessing a healthy diet, did the authors also explore potential enablers or factors that facilitate access? Understanding these enablers is crucial for designing targeted and cost-effective interventions, as solutions within the community may be more effective in addressing barriers.

Methods

- Instead of “written consent”, consider “written informed consent”.
- “to gather information from Key Informant Interviews (KIIs), …” or “to gather information through Key Informant Interviews (KIIs), …”?
- The study population was pregnant women LHIV aged 25 to 35 years in the study area. Why did the authors choose this age group? What were the inclusion and exclusion criteria used?
- The last sentence of the Study settings subsection state that “The selection of these councils was informed by the THIS 2022-2023 [15].” Could the authors clarify how the findings or recommendations from the THIS 2022-2023 report specifically influenced the selection of these councils for the study? What were the key factors or data from the report that guided this decision?
- The title for Table 1 could be improved from “**Distribution on the number of FGDs conducted**” to something like “**Distribution of FGDs Conducted**”. Similar improvements could be made to Table 2 etc.
- Each FGD session consisted of 7 to 12 participants. It would be helpful to include the duration of each session, specifying the time range (e.g., between X and Y minutes or hours).
- The statement below Table 2, “***22 interviews were conducted in urban settings, while 24 interviews took place in rural areas**,” is not clearly linked to the table. What does the asterisk (*) represent in this context? If urban and rural settings are important features of the study, it may be helpful to structure Table 2 to reflect these categories for better clarity.

**Discussion**

- The study seems to be missing a discussion on **enabling factors** that could improve access to nutritious foods for pregnant women living with HIV. For instance, the **Njombe region** is known for its **fertile and arable land**, along with favorable **weather conditions**, which provide significant opportunities to enhance the production of nutritious foods. Positioning the study within the context of these existing opportunities would not only strengthen the findings but also increase the broader applicability of the study, particularly in highlighting how local resources and conditions could help improve access to nutritious foods for pregnant women living with HIV. Addressing both barriers and enablers would offer a more comprehensive understanding and actionable steps for improving maternal nutrition in this specific context.
