# Supplementary material for: Exploring barriers to accessing healthy diets among pregnant women living with HIV in the Njombe region, Tanzania: A qualitative study
Source: PLOS Glob Public Health. 2025 Oct 10;5(10):e0004438. doi: 10.1371/journal.pgph.0004438 (PMC12513609; doi:10.1371/journal.pgph.0004438)
Supplement: Attachment — Submitted filename: A rebuttal letter_27_06_2025.doc [file pgph.0004438.s009.doc]

Dear Sarah Jose, Ph.D.

Thank you very much for giving us the opportunity to submit a revised draft of our manuscript titled “Barriers to accessing a healthy diet among HIV pregnant women in the Njombe region of Tanzania: A qualitative study”. We appreciate the time and effort that you and reviewers have dedicated to providing valuable feedback on our manuscript. Here is a point-by-point response to journal requirements and reviewers.

**Journal Requirements**:

**Comment 1**: We have noticed that you have uploaded Supporting Information files, but you have not included a list of legends. Please add a full list of legends for your Supporting Information files after the references list.

**Response**: The list of legends has been included after the references list (page 24).

**Comment 2**: Thank you for uploading your study's underlying data set. Unfortunately, the repository you have noted in your Data Availability statement does not qualify as an acceptable data repository according to PLOS's standards. At this time, please upload the minimal data set necessary to replicate your study's findings to a stable, public repository (such as figshare or Dryad) and provide us with the relevant URLs, DOIs, or accession numbers that may be used to access these data. For a list of recommended repositories and additional information on PLOS standards for data deposition, please see https://journals.plos.org/plosone/s/recommended-repositories
**Response**: The data availability statement has been revised (page 24).

**Review Comments to the Author**

**Reviewer #1**

**Comment 1**:

Overall, the authors should provide a more thorough justification for their study, particularly explaining why the Njombe region was selected as the study area. Additionally, the choice of the study population—pregnant women living with HIV aged 25-35 years—requires justification, as the typical reproductive age range is 15-49 years. This can be addressed by offering a clear description of the inclusion and exclusion criteria used. While the study focuses on barriers, it would benefit from also exploring enablers, as this would provide a more balanced perspective and broaden the study’s applicability. I have uploaded a file with my detailed review feedback for the authors.
**Response:** We have addressed the comments and provided justifications for selecting the Njombe region and the age group of 25 to 35 years (pages 5-6). Additionally, we have expanded the discussion section to include the enabling factors in the Njombe region (page 16). We sincerely appreciate the additional feedback you provided; we agreed with most of your comments and have incorporated them throughout the manuscript, including in the title, abstract, introduction, methods, and discussion sections.

**Reviewer #2
Comment 1:** The authors conducted a well-designed qualitative study that explored the barriers to accessing a healthy diet among HIV-positive pregnant women in the Njombe region of Tanzania. This study provides important context-specific insights into the unique nutritional challenges faced by HIV-infected pregnant women, a population often underrepresented in existing nutritional research literature. It has the potential to add great value to the understanding of individual, community, organizational, and environmental barriers. Overall, this paper offers valuable implications for maternal health interventions and policy development. However, there are some major concerns for the authors to consider:

As mentioned in the Introduction, existing studies have identified several barriers such as influence of family members, economic limitations, and food scarcity. It is yet not clear how this study uniquely contributes to filling a particular gap in the literature.
**Response:** We agreed with the comment and included information showing how the present work uniquely contributes to filling a specific gap in the literature (page 5).

**Comment 2**: I encourage the authors to further unpack what factors mattered more across different groups (e.g., do HIV pregnant women, women of reproductive age, and men had similar or different perceptions on the topics).

**Response**: We appreciate your insightful comments and have included an appendix titled ‘*S3 Thematic and key factors’* (page 9).

**Comment 3**: I suggest writing out the exact number of participants in each category in Table 1. It is confusing right now as it only shows the number of FGD sessions (which is 1 in all categories and not very informative).
**Response**: Table 1 has been revised as suggested (page 8).

**Comment 4**: The Results and Discussion sections were not very well structured. I appreciate the authors citing quotes from the participants but currently It is difficult to tell which factors were considered to be more important (or more often brought up) by which group of participants.

**Response**: Thank you for bringing this matter to our attention. We have included an appendix titled ‘*S3 Thematic and key factors’* (page 9), and hopefully it is now clear and easy to follow the results and discussion sections.

**Comment 5**: Although the study is frame worked as a qualitative research, it would be still valuable to have summary tables in the Results section to provide counts of different thematic themes and key factors.

**Response**: We value your feedback and have created a summary table of key themes and factors included an appendix titled ‘*S3 Thematic and key factors’* (page 9).

**Comment 6**: The Discussion was comprehensive, but it could be more directly tied back to the initial objectives and the research questions. Drawing out clear links between the findings and the broader context of maternal and HIV nutrition policies in Tanzania could make this piece more effectively demonstrate the implications and practical relevance of your results.

**Response:** Thank you for your feedback. We have added a paragraph connecting our findings to maternal and HIV nutrition policies in Tanzania (pages 21 and 22).

**Comment 7**: Related to the comment above, the Discussion section could benefit from an emphasis on implications for policy and practice.

**Response:** We have included a paragraph illustrating the connections between our findings and the broader context of maternal and HIV nutrition policies in Tanzania (pages 21 and 22).

**Reviewer #3**

**Comment 1**: The study is of significant relevance, as it addresses the barriers to healthy diets among HIV-positive pregnant women in Tanzania at a critical juncture. The application of ethnographic qualitative methods, including focus group discussions, in-depth interviews, and key informant interviews, with a diverse range of participants such as HIV-positive women, men, healthcare providers, and community leaders enhances the robustness of the study. Employing the socio-ecological model to categorize findings into individual, community, organizational, and environmental factors is executed proficiently. The connection between the findings and existing literature is effectively articulated, and the practical implications are clearly highlighted. Furthermore, the transparent declaration of ethical considerations, data availability, funding sources, and competing interests contributes positively to the overall integrity of the research. The author can try to reduce the words in the abstract as it is a bit too detailed. For PLOS Global Public Health, abstracts should be sharp and within the word limit (preferably under 300 words). Try to reduce it by focusing on major findings, key methods and conclusion. Generally, it is well researched pare and excellent work for the author.

**Response:** Thank you for agreeing to review our manuscript. We have verified the word count in the abstract section, which stands at 285 words, and we can confirm that it complies with the specified requirements (page 2).

Sincerely,

Ray Masumo, PhD
