# Supplementary material for: Exploring barriers to accessing healthy diets among pregnant women living with HIV in the Njombe region, Tanzania: A qualitative study
Source: PLOS Glob Public Health. 2025 Oct 10;5(10):e0004438. doi: 10.1371/journal.pgph.0004438 (PMC12513609; doi:10.1371/journal.pgph.0004438)
Supplement: Attachment — Submitted filename: Comments for the review.docx [file pgph.0004438.s008.docx]

Comments for the review of a manuscript titled: **Exploring barriers to accessing healthy diets among pregnant women**

This manuscript examines a critical public health issue: the barriers to healthy diets faced by pregnant women living with HIV in a high-burden region of Tanzania. The study is timely, relevant, and thoroughly grounded in both theoretical and contextual frameworks. Using the socio-ecological model and ethnographic methods helps develop a rich understanding of the complex barriers operating at individual, community, organizational, and environmental levels.

However, the manuscript can be improved through clearer language, deeper analysis in the discussion, and a stronger connection between the results and existing literature. These improvements will increase the manuscript’s impact and readability for a global audience.

**Major Comments**

Clarity and Language Use: The manuscript contains many grammatical errors and awkward sentences that sometimes hinder understanding. A thorough language and copyediting revision is strongly recommended to improve clarity and flow.

Integration with Literature: While the introduction is well-referenced, the discussion lacks sufficient synthesis with regional and global literature. The authors should better explain how their findings relate to, or differ from, previous studies on maternal nutrition in HIV-affected populations.

- Analytical Depth: The results are mostly descriptive. The discussion should provide deeper analysis and interpretation, especially in linking findings to larger structural factors such as poverty, gender norms, and health system capacity.
- Presentation of Qualitative Data: Consider including more contextual details about response frequency (e.g., “many participants,” “a few participants”) to help readers understand the weight of different themes. A summary table based on the socio-ecological model would also serve as a helpful visual.
- Nutrition-Specific Outcomes: The study describes general barriers but could explore specific nutritional issues, such as micronutrient deficiencies, particularly given the higher nutritional needs of pregnant women living with HIV.
- Limitations Section: The limitations should be expanded to mention potential recall bias, social desirability bias (especially regarding stigma or alcohol use), and limitations in applying the findings beyond the Njombe region.

**Minor Comments**

- Terminology Consistency: Some terms (e.g., “appended” vs. “applied,” “knowledge capacity”) need standardization and clearer definitions.
- Reference Formatting: Please review the reference style for consistency with journal guidelines.
- Formatting of Tables: Tables 1 and 2 are informative but could benefit from clearer titles and better alignment for ease of reading.
- Spelling/Typos: A few typos are present (e.g., "que" should be "queue," and "3023" instead of "2023" for dates).
- Figure Suggestion: Consider adding a conceptual figure illustrating barriers at each level of the socio-ecological model.

**Recommendation: Minor Revision**

This paper addresses a vital topic with significant policy and program implications. While the study design and framework are solid, the manuscript needs editing and some moderate improvements in analysis and discussion. With these changes, the paper will make a valuable contribution to the journal.bution to the journal.
