# Supplementary material for: Exploring barriers to accessing healthy diets among pregnant women living with HIV in the Njombe region, Tanzania: A qualitative study
Source: PLOS Glob Public Health. 2025 Oct 10;5(10):e0004438. doi: 10.1371/journal.pgph.0004438 (PMC12513609; doi:10.1371/journal.pgph.0004438)
Supplement: Attachment — Submitted filename: A rebuttal letter_25_08_2025.doc [file pgph.0004438.s010.doc]

Dear Miho Sato

Thank you for giving us the opportunity to submit a revised draft of our manuscript titled “Exploring barriers to accessing healthy diets among pregnant women living with HIV in the Njombe region, Tanzania: A qualitative study.” We sincerely appreciate the time and effort you and the reviewers have dedicated to providing valuable feedback on our manuscript. Enclosed is a detailed point-by-point response addressing the reviewers’ comments and suggestions.

**Journal Requirements**

**Comment 1:** If the reviewer comments include a recommendation to cite specific previously published works, please review and evaluate these publications to determine whether they are relevant and should be cited. There is no requirement to cite these works unless the editor has indicated otherwise. .
**Response**: We have reviewed and evaluated all publications, making corrections to ensure their relevance.

**Comment 2:** Please review your reference list to ensure that it is complete and correct. If you have cited papers that have been retracted, please include the rationale for doing so in the manuscript text, or remove these references and replace them with relevant current references. Any changes to the reference list should be mentioned in the rebuttal letter that accompanies your revised manuscript. If you need to cite a retracted article, indicate the article’s retracted status in the References list and also include a citation and full reference for the retraction notice.

**Response**: We reviewed the reference list and made minor corrections for relevance.

**Review Comments to the Author**

**Reviewer #1**

**Comment 1**: The authors have addressed my previous comments, and I commend them for their efforts. I have no further comments, except for one minor observation: the word "appexed" appears to be a typographical error and should likely be corrected to "appended".
**Response:** Thank you for your insightful suggestions and recommendations. We have corrected the words as needed.

**Reviewer #2
Comment 1**: All comments have been addressed. I appreciate the authors adding the important content in discussion as well as appendix table.

**Response:** Thank you very much for your valuable suggestions and recommendations.

**Reviewer #3**

**Comment 1:** Clarity and Language Use:

The manuscript contains many grammatical errors and awkward sentences that sometimes hinder understanding. A thorough language and copyediting revision is strongly recommended to improve clarity and flow

**Response:** Thank you for bringing this to our attention. We have conducted a thorough revision of the language.

**Comment 2**:

Integration with Literature: While the introduction is well-referenced, the discussion lacks sufficient synthesis with regional and global literature. The authors should better explain how their findings relate to, or differ from, previous studies on maternal nutrition in HIV-affected populations.

**Response:** We sincerely appreciate your feedback. Corrections have been made in the discussion based on your suggestions.

**Comment 3:** Analytical Depth: The results are mostly descriptive. The discussion should provide deeper analysis and interpretation, especially in linking findings to larger structural factors such as poverty, gender norms, and health system capacity.

**Response:** Thank you for bringing this to our attention. We have revised the discussion section as you suggested.

**Comment 4**: Presentation of Qualitative Data: Consider including more contextual details about response frequency (e.g., “many participants,” “a few participants”) to help readers understand the weight of different themes. A summary table based on the socio-ecological model would also serve as a helpful visual.

**Response:** We agreed with the comment and restructured our qualitative data presentation as suggested.

**Comment 5**: Nutrition-Specific Outcomes: The study describes general barriers but could explore specific nutritional issues, such as micronutrient deficiencies, particularly given the higher nutritional needs of pregnant women living with HIV.

**Response:** We have included this in the discussion of study limitations.

**Comment 6**: Limitations Section: The limitations should be expanded to mention potential recall bias, social desirability bias (especially regarding stigma or alcohol use), and limitations in applying the findings beyond the Njombe region.

**Response:** We have expanded to address potential recall bias, social desirability bias, and limitations in generalizing the findings beyond the Njombe region.

**Minor Comment**:

**Comment 1:** Terminology Consistency: Some terms (e.g., “appended” vs. “applied,” “knowledge capacity”) need standardization and clearer definitions.

**Response:** We have made the appropriate corrections in accordance with your suggestions. **Comment 2**: Reference Formatting: Please review the reference style for consistency with journal guidelines.

**Response:** We have made the appropriate corrections in accordance with your suggestions..

**Comment 3**:	Formatting of Tables: Tables 1 and 2 are informative but could benefit from clearer titles and better alignment for ease of reading.
**Response:** Thank you for your feedback; we have made the necessary corrections based on your suggestions.
**Comment 4**: Spelling/Typos: A few typos are present (e.g., "que" should be "queue," and "3023" instead of "2023" for dates).

**Response:** We have made the appropriate corrections in accordance with your suggestions.

**Comment 5**: Figure Suggestion: Consider adding a conceptual figure illustrating barriers at each level of the socio-ecological model.

**Response:**

We have created a conceptual figure illustrating barriers at each level of the socio-ecological model.

Sincerely,

Ray Masumo, Ph.D.
